# Biodegradable Natural Rubber Based on Novel Double Dynamic Covalent Cross-Linking

**DOI:** 10.3390/polym14071380

**Published:** 2022-03-29

**Authors:** Qinggeng Jiang, Yi Gao, Lusheng Liao, Rentong Yu, Jianhe Liao

**Affiliations:** 1 School of Materials Science and Engineering, Hainan University, Haikou 570228, China; qinggeng_j@163.com (Q.J.); yii_gao@163.com (Y.G.); 990359@hainanu.edu.cn (J.L.); 2 Guangdong Provincial Key Laboratory of Natural Rubber Processing, Agricultural Products Processing Research Institute of Chinese Academy of Tropical Agricultural Sciences, Zhanjiang 524001, China; lsliao@catas.cn

**Keywords:** epoxidized natural rubber, cyclic carbonate, biodegradable, dual cross-linked network, self-healing

## Abstract

In this paper, biodegradable epoxidized natural rubber containing cyclic carbonate groups (CNR) was prepared by the reaction between epoxidized natural rubber (ENR) and carbon dioxide. Dynamic disulfide bonds and a boronic ester structure were successfully constructed and then the cross-linking network was formed by the thermally initiated “click” reaction between thiol groups of the cross-linker and the residual epoxy groups of ENR. As a result of the exquisite double dynamic covalent structure, the material exhibits high self-healing efficiency. Moreover, by virtue of the cyclic carbonate structure of the CNR, the natural rubber was confirmed to be biodegradable according to the biodegradable measurement. To the best of our knowledge, natural rubber with biodegradable and self-healing characteristics was obtained for the first time.

## 1. Introduction

Natural rubber (NR), compared with non-renewable fossil-based materials, is also a sustainable polymer material. NR has been widely used in the tire industry, as well as the aerospace and medical fields, owing to its unique features of high tensile and tearing strength, high fatigue resistance, low heat built-up and so on [1]. After curing, the covalent cross-linked structure endows NR with excellent comprehensive properties; however, the crosslinked NR would be “insoluble” and “unmeltable”, and could not be recycled without impairment of performance, leading to crucial black pollution consequences for the environment [2]. It would be of significance to reuse the vulcanized rubber products for the purpose of alleviating the burden on the Earth. To achieve secondary utilization of NR, some technologies, such as a hydropress with a liquid atmosphere, microwave or ultrasonic waves assistance, and regenerating agents have been employed [2,3]. In these ways, waste rubber could be reprocessed with the sacrifice of mechanical properties and energy consumption. Recently, a novel and powerful self-healing strategy utilizing dynamic covalent bonds was proposed [4,5,6,7].

A dynamic covalent bond is a kind of chemical bond which can be physical cross-linked and is dynamically reversible. It can be dissociated and reconstructed under the stimulation of mild conditions or thermal-light conditions, as well as exchange reactions, which has recently been identified as an extremely important kind of chemical bond [8,9]. Reversible covalent bonds usually exhibit higher bond strength and better stability than non-covalent bonds, and therefore they have broad application prospects in dynamic polymers, especially in the structural design of materials [10,11]. A considerable number of exchange reactions, including disulfide exchange [12,13], boronic ester exchange [14,15,16,17], Diels-Alder chemistry [18,19], siloxane equilibration [20], imine exchange [21,22], transesterification [23,24], and silyl ether exchange [25,26] in the context of dynamic polymers have been developed to improve the efficiency of service.

This dazzling field has attracted the notice of many research groups. For example, Santana et al. [27] first introduced furan functional groups into natural rubber and used the Diels-Alder coupling reaction to react furfurylamine with maleic anhydride groups to form furan functional group imide, which was grafted onto the NR skeleton to form maleic anhydride grafted rubber (NR-*g*-MA). The material exhibited self-healing behavior at 130 °C, especially at low strain. After self-healing, the tensile strength of the samples can reach 80% of the original. Guo et al. [28] also achieved styrene-butadiene rubber (SBR) with a self-healing function. The vitrified rubber was synthesized by the thiol-ene click reaction of dithiol-containing boronic ester with SBR. Due to the transesterification of boronic ester bond, the network topology can be changed, and thus the material was provided with a self-healing ability and ductility. To improve self-healing efficiency, Yang et al. [6] reported epoxidized natural rubber cross-linked by a series of aromatic disulfides and double cross-linked by dynamic disulfide decomposition and thermal reversible hydrogen bonds. The self-healing efficiency of double cross-linked ENR was as high as 98%, and simultaneously fracture or cyclic fatigue damage had no negative effect on the self-healing property of the material.

**Scheme 1:**
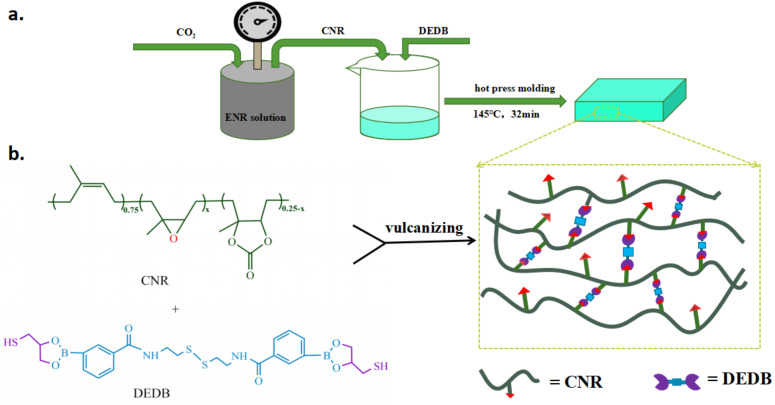
(**a**) Formation of cyclic carbonate groups and the double dynamic covalent network; (**b**) Generation of CNR-DEDB linkage *via* thermally initiated thiols and epoxy groups of “click” reaction.

Although a large number of articles have reported self-healing rubber with high self-healing efficiency, its self-healing ability will gradually weaken with the increase of the number of self-healing cycles, and became waste rubber. As far as we know, natural rubber that is biodegradable and self-healing has so far not been reported on. In this work, we used the epoxy group of ENR to react with carbon dioxide to obtain a weak bond of cyclic carbonate, which was intended to make the rubber (CNR) biodegradable. In addition, we constructed units of disulfide bond and boronic ester for the purpose of a dynamic reversible effect. Because of the dynamic characteristics of boronic esters and disulfide bonds, the cross-linked network of the rubber can be rearranged, which endowed CNR with the ability of self-healing. The experimental process was shown in Scheme 1. The ENR with both biodegradability and self-healing functions was thus produced, which greatly reduced the negative impact of tradition natural rubber materials on the environment.

## 2. Materials and Methods

### 2.1. Materials

ENR was obtained from the Agricultural Products Processing Research Institute, Chinese Academy of Tropical Agricultural Sciences, with an epoxy content of 25 mol%. Carbon dioxide (CO_2_, 99.99%) was purchased from Hainan Jiateng Chemical Gas Co., Ltd. (Haikou, China). Tetrabutylammonium iodide (TBAI, 98%); cystamine dihydrochloride and deuterium chloroform were supplied by J&K Scientific Co., Ltd. (Beijing China). N-hydroxysuccinimide (NHS), 1-thioglycerol, 1-ethyl-3-(3-(dimethylamino)propyl) carbodiimide hydrochloride (EDC), and phosphate buffer solution (PBS) were supplied by Shanghai Macklin Biochemical Co., Ltd (Shanghai China). Deionized water was produced with an ultrapure water machine. Solvents such as toluene were analytical reagent grade without further treatment. All other reagents were purchased from Xilong Technology Co., Ltd (Shantou, China). 

### 2.2. Methods

#### 2.2.1. Preparation of Carbonated Natural Rubber (CNR)

**Scheme 2:**
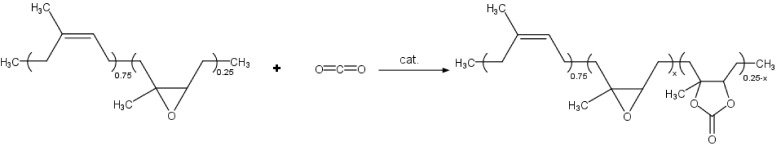
Synthesis of CNR from the reaction of ENR and CO_2_ with the catalyst of TBAI.

CNR was synthesized according to Scheme 2. A rubber solution was prepared according to Gan et al.’s work [29]. 10 g ENR was masticated by an open mixer (LN-6, Guangdong Lina Industrial Co., Ltd., Dongguang, China). The roll diameter was 16 cm, and the gap between the two rolls was adjusted to 0.1 mm. After grinding the ENR for 20 min, the ENR was dissolved in toluene in a flask for 7 days (30 g ENR dissolved in 100 mL toluene) to allow it to be dissolved sufficiently. The CNR was synthesized by coupling CO_2_ with 4 g ENR in a 25 mL autoclave (YZPR-500ML, Shanghai Yanzheng Experimental Instrument Co., Ltd, Shanghai, China). The amount of catalyst was 10% with respect to ENR. After 5 min of leakage test and purification with CO_2_, the ENR solution was stirred in a CO_2_ atmosphere and heated to 130 °C at a ramp rate of 10 °C/min. Reaction time parameters of 12 and 30 h and reaction pressure parameters of 0.5 and 1 MPa were adopted orthogonally for the sake of obtaining the CNR with high cyclic carbonate conversion. After the reaction, the viscous solution was transferred to a rotary evaporator in consideration of concentration. Then, the condensed solution was poured into a sufficient amount of methanol for precipitation. Afterward, the product was dried in *vacuo* at 70 °C for 24 h. The corresponding CNR was abbreviated as CNR-0.5 M12 h, CNR-1M12 h, CNR-0.5M30 h, and CNR-1M30 h, respectively.

#### 2.2.2. Synthesis of N,N’-(Disulfanediylbis(ethane-2,1-diyl))bis(3–4-(mercaptomethyl)-1,3,2-dioaboroborolan-2-yl)benzaminde) (DEDB)

**Scheme 3:**
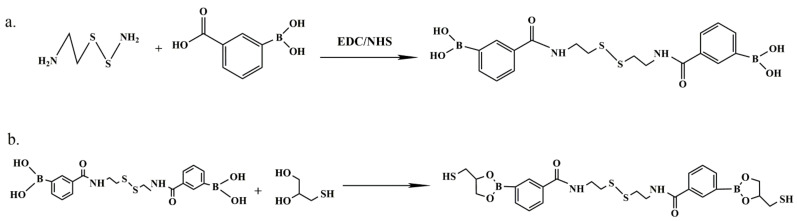
(**a**) Synthesis of BPBAC *via* the EDC/NHS coupling reaction between CPBA and cystamine dihydrochloride; (**b**) Synthesis of DEDB from the reaction of BPBACwith 1-thioglycerol.

Firstly, as shown in Scheme 3a, the cross-linker Bis(phenylboronic acid carbamoyl) Cystamine (BPBAC) was prepared by a coupling reaction between 3-carboxylphenylboronic acid (CPBA) and cystamine dihydrochloride. NHS (1.40 g, 12.20 mmol), EDC (1.53 g, 8.00 mmol) and CPBA (1.65 g, 10.00 mmol) were dissolved in 20 mL PBS (pH = 6.80) and stirred for 2 h, after which cystamine dihydrochloride (0.90 g, 4.00 mmol) was added. The reaction mixture was stirred at room temperature for 12 h, and the obtained solution was filtered under vacuum. After that, BPBAC was rinsed with deionized water, recrystallized with methanol, and finally dried in a vacuum oven. Finally, the crosslinking agent DEDA, a disulfide-containing boronic ester, was obtained from the reaction of BPBAC and 1-thioglycerol, as shown in Scheme 3b. Briefly, BPBAC (1.35 g, 3.01 mmol) was dissolved in 15 mL deionized water until it was completely dissolved. Then, 1-thioglycerol (1.00 g, 9.28 mmol) was added and stirred for 5 min, after which magnesium sulfate (MgSO_4_, 1.25 g) was added to react at room temperature for 24 h. After the reaction was completed, the resulting product was filtered, and then the filtrate was concentrated. Finally, the product was kept drying in a 100 °C vacuum oven for 24 h.

#### 2.2.3. Procedure for CNR Cross-Linking

Firstly, 600 mg of the cross-linker was added to the homogeneous solution containing 60 g CNR and 200 mL toluene, and the mixture was stirred continuously at room temperature for 8 h. The viscous solution was concentrated by a rotary evaporator, and then the concentrated solution was dropped into excessive methanol for precipitation. The product featured with a dynamic cross-linked polymer network was put in a vacuum oven and dried at 60 °C for 48 h to further remove the residual solvent. Then, the resultant rubber material was hot pressed. The vulcanizing temperature was 145 °C and the curing time was 21 min, which was obtained by a rubber processing analyzer (RPA).

#### 2.2.4. Characterizations

FTIR. Fourier transform infrared spectra (FTIR) were collected with 32 scans and a resolution of 4 cm^−1^ in a FTIR spectrometer (Spectrum One FTIR, PerkinElmer, Shelton, CT, USA) with attenuated total reflectance (ATR) mode.

^1^H NMR. Nuclear magnetic resonance spectra (^1^H NMR) were collected on a spectrometer (AV 400 NMR, Bruker, Billerica, MA, USA) at 400 MHZ and 25 °C. Deuterated water (D_2_O) and deuterated chloroform (CDCl_3_) were used as solvents, and tetramethylsilane (TMS) was used as internal standard.

TGA. Thermal gravimetric analysis (TGA) was conducted by a thermogravimetric analysis (SDT Q600, TA Instruments, Delaware, USA) The specimen was heated from 25 °C to 500 °C under a nitrogen atmosphere at a rate of 10 °C/min.

DSC. Differential scanning calorimetry (DSC) was measured on a instrument (Toledo DSC 822e, Mettler, Zurich, Switzerland). A 5–10 mg sample was heated to 20 °C to remove the thermal history, then cooled to −20 °C, and then heated to 20 °C on an aluminum pan to evaluate the glass transition temperature (*T*_g_) value. The heating rate was 10 K/min, and the whole process was carried out in a nitrogen atmosphere with a flow rate of 10 mL/min.

WCA. The water contact angle (WCA) was measured by a instrument (SDC-100, SINDIN, Dongguang, China) contact angle analyzer as averages of 5 measurements.

Tensile measurement. The tensile properties of CNR were tested according to ISO 37-2017 using a electronic universal testing machine (CMT 4104, SANS, Shenzhen, China) instrument at room temperature with a 500 mm/min strain rate.

Self-Healing tests. The self-healing measurements were carried out by cutting the test sample into two completely separated pieces with a blade and then keeping the two pieces connected gently to ensure the cut surfaces fitted entirely at 60 °C for 12 h, 24 h, and 48 h, respectively. The repaired specimens were measured using standard static uniaxial tensile tests, and the mechanical properties of each sample were measured three times. The self-healing efficiency was determined from the Equation (1):

(1) $$H_{\mathrm{self}-\mathrm{healing}}=\frac{\sigma_{\mathrm{healed}}}{\sigma_{\mathrm{pristine}}}\times 100\%$$

where *H*_self-healing_ is the efficiency of self-healing and *σ*_pristine_ and *σ*_healed_ are the maximum stress reached during stretching of the original sample and self-healing sample, respectively. 

Biodegradable tests. The biodegradable performance was analyzed by weight loss. CNR-DEDB samples in the shape of a square with the size of 1 cm × 1 cm and the weight of *W*_0_ were placed in 30 mL glass bottles containing PBS (pH = 7.4) for 28 days. PBS was replaced every 4 days and the weight *W*_t_ was recorded every 4 days. Biodegradation efficiency was evaluated from the Equation (2):

(2) $$\;H_{biodegradation}=\frac{W_{0}-W_{t}}{W_{0}}\times 100\%$$

where *H_biodegradation_* is the efficiency of biodegradation, *W*_0_ is the initial sample weight, and *W_t_* is the sample weight at a given time. 

## 3. Results and Discussion

### 3.1. Physicochemical Structure of CNR

**Figure 1:**
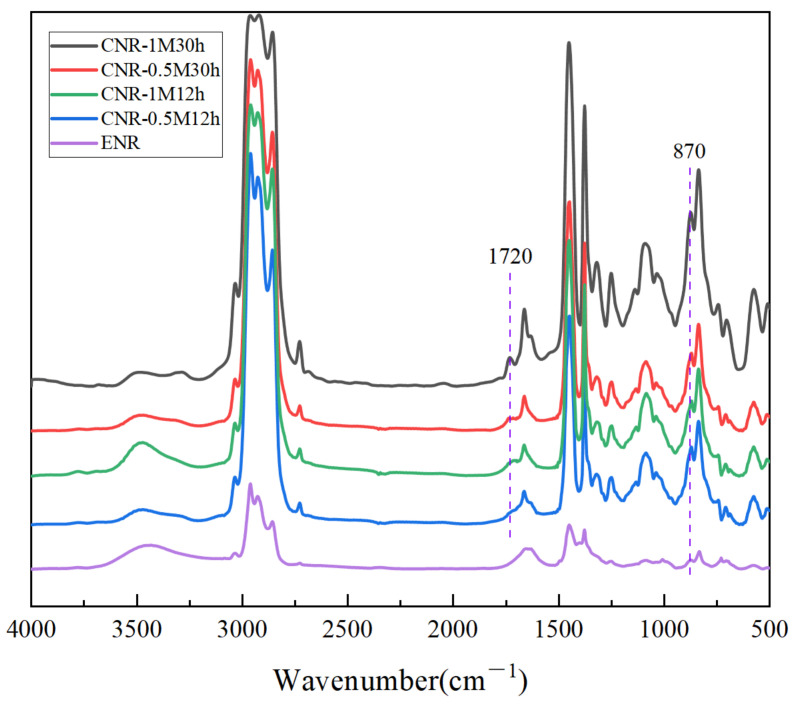
FTIR spectra of ENR and CNR under different reaction pressures and reaction time.

From Figure 1, the spectra clearly showed the structural difference between ENR and CNR. The results showed that a new peak appeared at 1720 cm^−1^, which belonged to C=O stretching vibration of the carbonate units. The epoxy -O- signal peak at 870 cm^−1^ was not completely faded, which was caused by the fact that the conversion of this reaction for the sample of 1M30 h reached maximum but not 100%, or the in-plane flexural vibration overlapped that of the -CH_3_ groups [30]. It was proved by FTIR that the epoxy groups were partly converted into cyclic carbonate groups.

**Figure 2:**
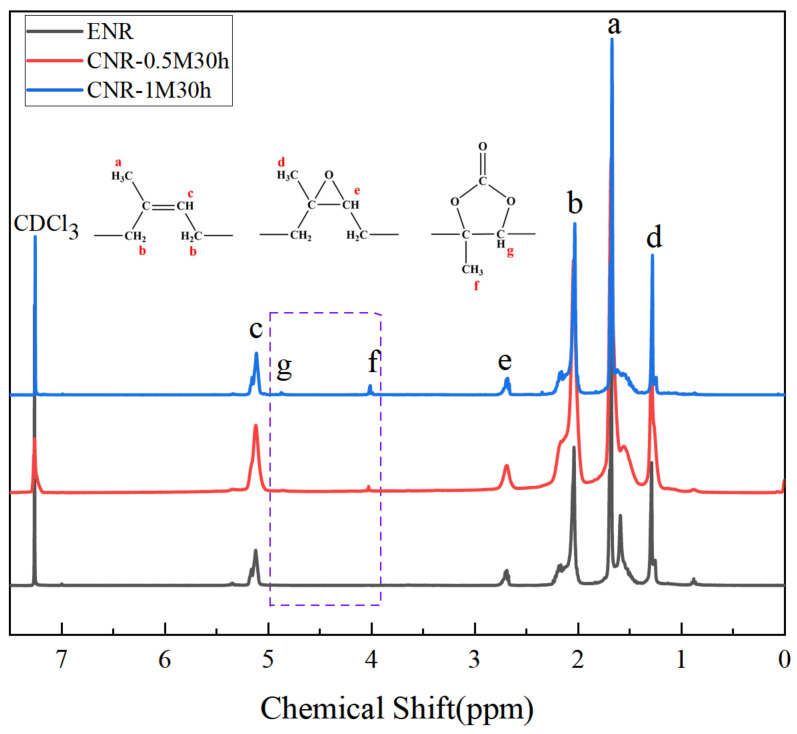
^1^H NMR spectra of ENR, CNR-0.5M30 h and 1M30 h.

Figure 2 illustrated the ^1^H NMR spectra of ENR, CNR-0.5M30 h, and CNR-1M30 h. Two new signals were found in the CNR ^1^H NMR spectrum at 4.0 and 4.8 ppm, respectively, and these two signals did not appear in the ENR spectrum. This also verified the formation of cyclic carbonates in CNR, which was consistent with the results of FTIR. According to Furst’s [31,32] method, the ^1^H chemical shift of cyclic carbonate group of 4,4,5-trimethyl-1,3-dioxolan-2-one was 4.0 ppm. Compared with Kawahara’s [33] propylene carbonate model, the two signals of 4.0 and 4.6 ppm were derived from the equatorial methylene and axial methylene of the cyclic carbonate group interacting with -CH_3_ and -H, respectively. The signal of CNR at 4.1 ppm could be attributed to the methyl proton of the cyclic carbonate group of CNR. In addition, by using the internal standard method in the ^1^H NMR of CNR-0.5M30 h and CNR-1M30 h, the conversions from epoxy groups to cyclic carbonate groups were 35% and 51%, respectively.

**Figure 3:**
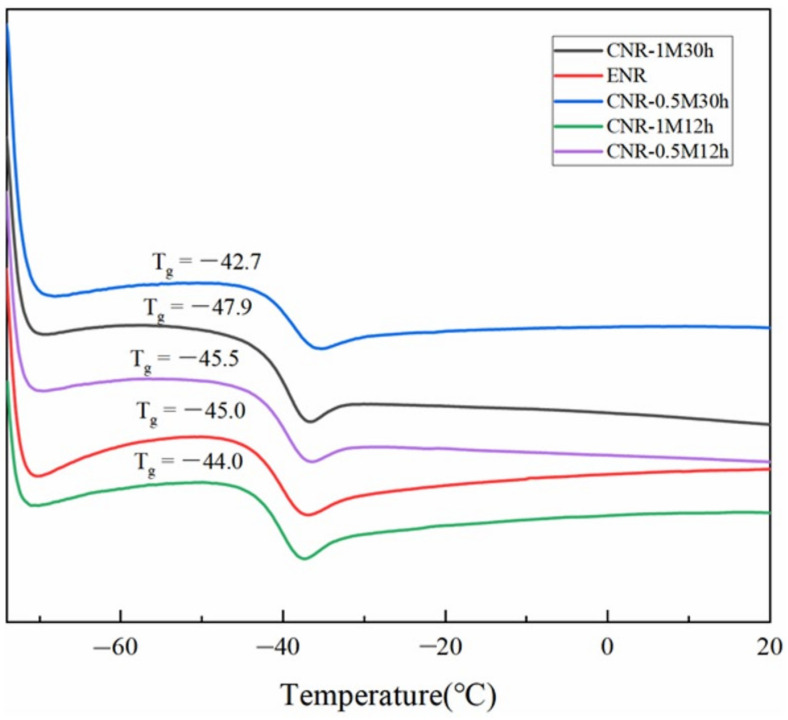
DSC curves of CNR with various reaction pressure and time compared with ENR.

The change of chemical structure was further investigated by DSC in Figure 3. According to the glass transition temperature measured by DSC, all the *T*_g_ values were still significantly lower than room temperature, indicating that CNR was still in a high elastic state at room temperature. In addition, the effect of reaction pressure or reaction time on the glass transition temperature of CNR seemed to be insensitive. In this sense, the five membered ring (cyclic carbonate structure) and the ternary ring (epoxy structure) had a similar influence on the segmental motion of the polymer chain, and the cyclic carbonate structure may be formed randomly across the ENR skeleton.

### 3.2. Preparation and Analysis of Cross-Linker DEDB

**Figure 4:**
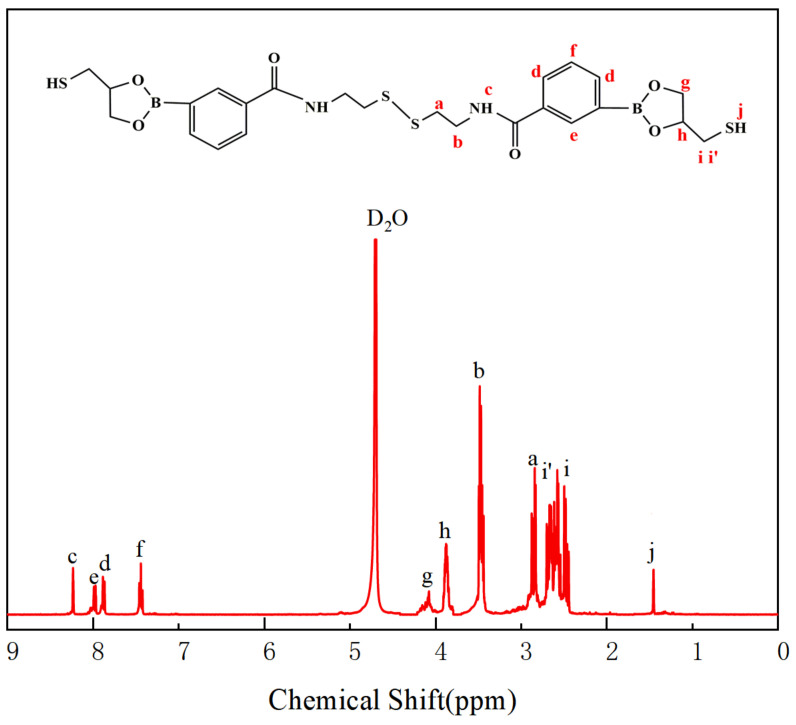
^1^H NMR spectrum of DEDB.

The synthetic procedure for the cross-linker DEDB is presented in Scheme 2. The peak value of ^1^H NMR of DEDB was shown in Figure 4: δ 2.84 ppm (t, 4H, -S-CH_2_- in position a), δ 3.50 ppm (t, 4H, -CH_2_-NH in position b), δ 8.23 ppm (s, 2H, -NH- in position c), δ 7.88 ppm (d, 4H, phenyl proton in position d), δ 8.00 ppm (d, 2H, phenyl proton in position e), δ 7.44 ppm (t, 2H, phenyl proton in position f), δ 4.10 ppm (m, 4H, methylene in position g), δ 3.87 ppm (t, 2H, methine in position h), δ 2.47 ppm (m, 2H, -S-CH_2_- in position i), δ 2.59 ppm (dd, 2H, -CH_2_-SH in position i′), and δ 1.45 ppm (s, 2H, -SH in position j). It can be confirmed that DEDB was successfully synthesized.

### 3.3. Preparation and Characterization of CNR-DEDB Elastomer

According to the FTIR and ^1^H NMR results of the reaction products obtained under different reaction conditions of press and time, the conversion of 1M30 h was the highest. For this reason, the sample of 1M30 h was investigated further. 

**Figure 5:**
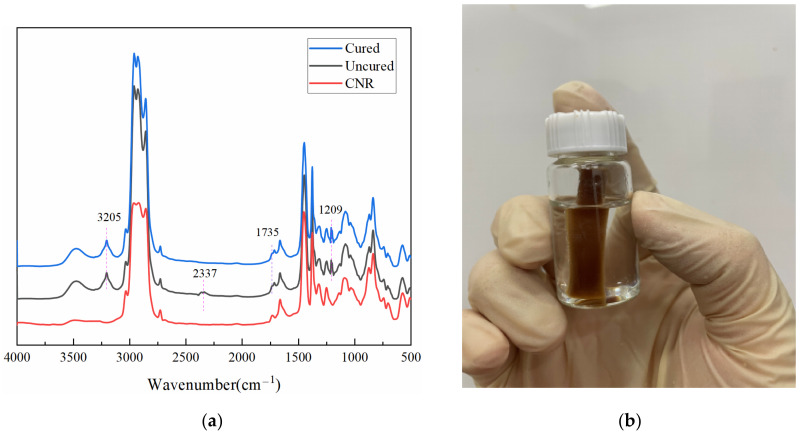
(**a**) FTIR spectra of CNR, uncured CNR-DEDB, and cured CNR-DEDB; (**b**) Photographs of CNR-DEDB after 72 h in toluene.

For the FTIR of CNR, chemical structure information of unvulcanized and vulcanized CNR-DEDB was shown in Figure 5a. Compared with CNR, new peaks at 1209, 1735, and 3205 cm^−1^ appeared in CNR-DEDB, which were caused by the stretching vibration of -SH, -BO and -NH of DEDB, respectively. Moreover, by comparing the FTIR spectra of unvulcanized and vulcanized CNR-DEDB, there was a peak at 2337 cm^−1^ caused by -SH stretching vibration when unvulcanized. However, this peak disappeared after vulcanization. Therefore, it was validated that the epoxy group reacted with the -SH group successfully by click reaction, and DEDB was successfully cross-linked with CNR. In addition, the CNR-DEDB did not dissolve at all after being immersed in toluene solution for 72 h, which further proved the success of cross-linking as shown in Figure 5b.

**Figure 6:**
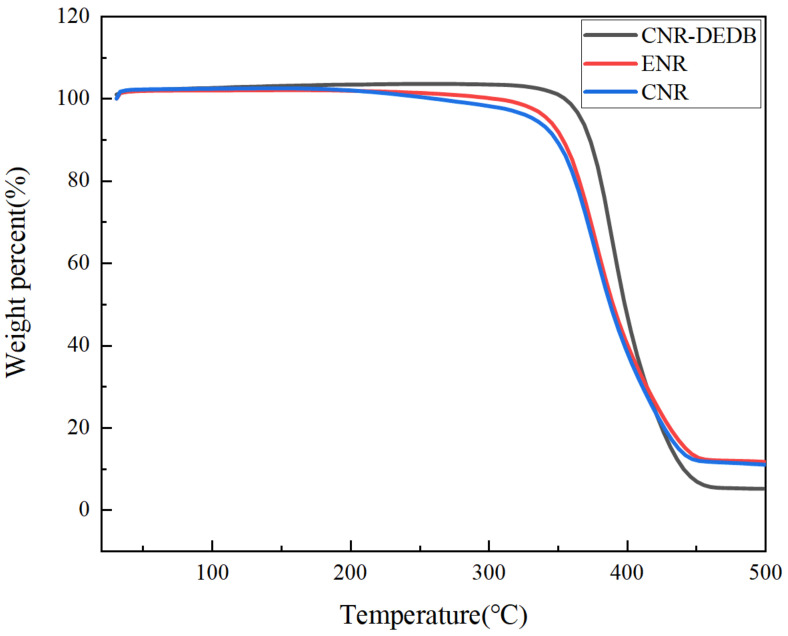
TGA curves of ENR, CNR and CNR-DEDB.

In order to verify the effect of cyclic carbonate groups and DEDBon thermal stability of ENR after cross-linking, TGA was used to analyze this performance of ENR, CNR, and CNR-DEDB. It was found that the formation of cyclic carbonate groups decreased thermal stability, as shown in Figure 6. With the DEDB cross-linking with CNR, the thermal decomposition temperature of CNR-DEDB increased compared with CNR. As shown in Table 1, the thermal decomposition temperature of CNR-DEDB was the highest, which was 363.96 °C for *T*_d-5%_ and 464.32 °C for *T*_d-max_ (*T*_d-5%_ is 5% weight loss temperature, and *T*_d-max_ is the maximum weight loss temperature). The raise of thermal decomposition temperature was interpreted to mean that the cross-linking inhibited the thermal degradation of CNR-DEDB, and thus improved the thermal stability of CNR-DEDB.

### 3.4. Self-Healing Procedure

**Figure 7:**
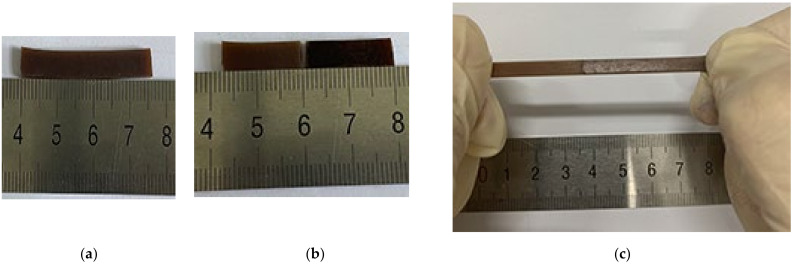
Photographs of self-healing of cut-off samples. (**a**) uncut; (**b**) cut; (**c**) self-healing.

The self-healing efficiency could be affected by healing time [4,6]. By controlling the healing time at the same repair temperature, the relationship between the repair efficiency and the healing time of the samples was explored. As shown in Figure 7, CNR-DEDB was cut into two parts, and one part was blackened while the other was brown. After healing at 60 °C for 12 h, it was obvious that the black part and the brown part had been partially healed.

**Figure 8:**
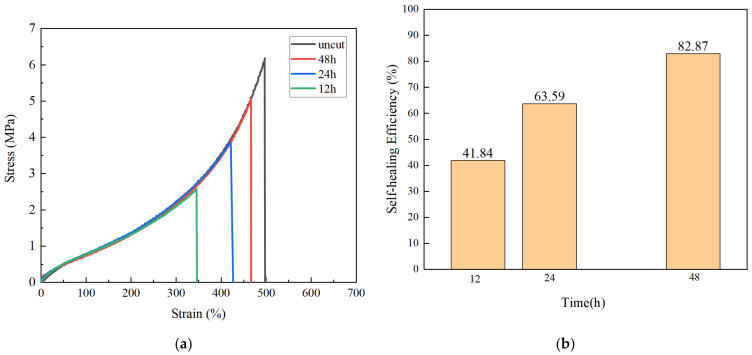
(**a**) Tensile curves of uncut sample and the CNR-DEDB elastomers with the self-healing time of 12 h, 24 h, and 48 h; (**b**) Self-healing efficiencies of the CNR-DEDB healed for 12 h, 24 h, and 48 h.

**Figure 9:**
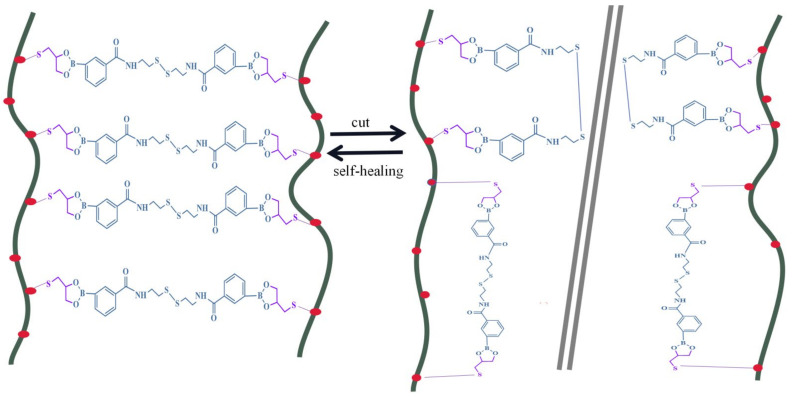
The mechanism of dynamic exchange of disulfide bonds and boronic ester.

In order to quantitatively acquire the healing degree, the self-healing degrees of samples healed for 12 h, 24 h, and 48 h were tested at a constant temperature of 60 °C, respectively. Figure 8a showed the difference of CNR-DEDB with a self-healing time from 12 to 48 h, which the tensile characteristics of the repaired specimens were closer to the tensile properties of the original sample, indicating that the efficiency of self-healing increased with the increase of sample repair time. From Figure 8b, when the repair time was prolonged, the degree of healing increased accordingly. Due to the transesterification of boronic ester bonds and disulfide exchange, the bond reorganization and rearrangement would occur, and the covalent bond would be re-established at the interface of the fracture surface of rubber, as shown in Figure 9 [4,6]. Therefore, more new covalent bonds could be created at the interface with the extension of repairing time. The reaction of the transesterification of boronic ester bonds and disulfide exchange was more complete, and the damaged parts would be repaired fully. After 12 h, the self-healing efficiency reached 41.84%. With the extension of time, the self-healing efficiencies of the samples continued to increase, but the tend of increase was significantly weakened. This resulted from the fact that the disulfide exchange and boronic ester exchange reaction between the segments reached equilibrium after a certain time, and the extension time was not as efficient as before.

### 3.5. Biodegradation Test

**Figure 10:**
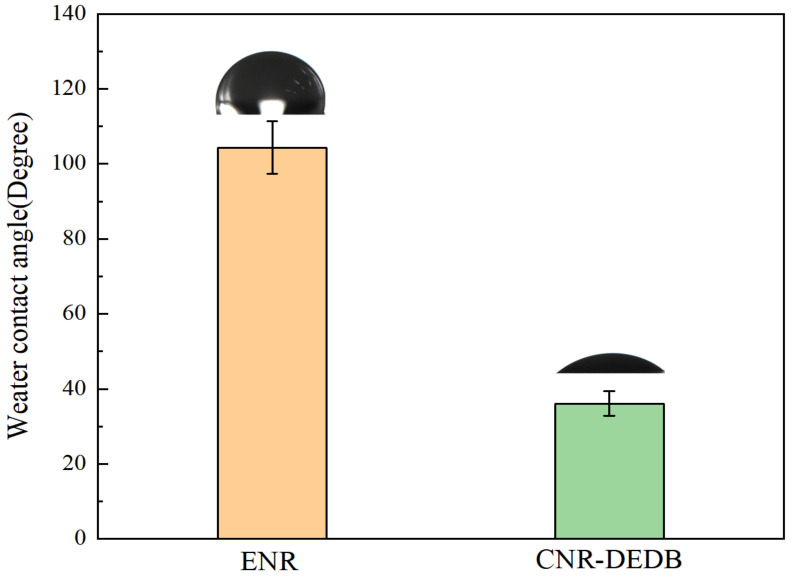
WCA of ENR and CNR-DEDB.

In general, the water absorption of polymer materials in soil burial or soaking process plays an important role in biodegradability. The higher the water absorption, the stronger the biodegradability will be. The water absorption of the tested sample is usually correlated with hydrophilicity [34]. Here, we analyzed the hydrophilicity of ENR and CNR-DEDB by testing the WCA. The smaller WCA was, the stronger hydrophilicity and the stronger biodegradability would be. Figure 10 exhibited that the WCA of ENR was about 104.3 degrees, indicating that it was hydrophobic. After cross-linking, the water contact angle decreased to 36.2 degrees, indicating a significant increase in hydrophilicity. This might be due to the high hydrophilicity of the CNR-DEDB containing cyclic carbonate group. As a consequence, the biodegradation ability of CNR-DEDB should be improved in comparison to ENR.

**Figure 11:**
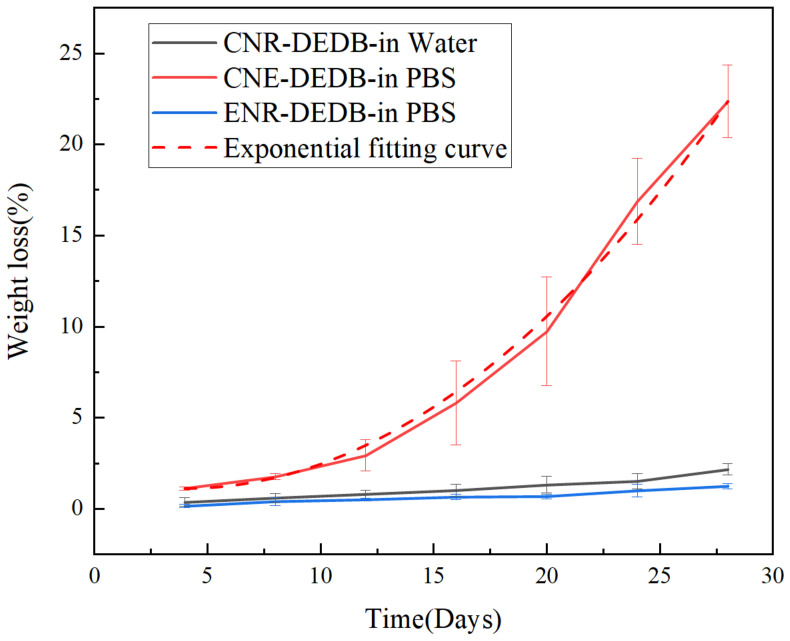
Weight loss ratio of CNR after soaking in PBS and water for 28 days, Weight loss ratio of ENR after soaking in PBS for 28 days and Exponential fitting curve of weight loss process of samples after soaking in PBS.

In this study, the CNR-DEDB elastomers were immersed in water or PBS for 28 days to measure its weight loss. The results shown in Figure 11 demonstrate that after soaking for 28 days, the weight loss rates of ENR-DEDB in PBS, CNR-DEDB in water, and PBS were 1.25%, 2.17%, and 22.37%, respectively. The weight loss rate of CNR-DEDB in PBS was much faster than that of hydrolysis. In fact, weight loss evaluation reflects performance of water resistance as an indirect measure of biodegradability. By comparing the weight loss behavior of CNR-DEDB in PBS and water, we can deduce that the faster weight loss resulted from the combined effect of hydrolysis and biodegradation. It is generally accepted that hydrolysis is one of the initial processes of biodegradation [35]. In a typical enzymatic degradation measurement, trace amounts of lipase will be incorporated into PBS. In this work, ENR was derived from natural rubber, a natural product containing lipase. As a result, weight loss of CNR-DEDB in PBS was obviously faster than that in water. In order to study the degradation behavior under soaking time, the weight loss curve was exponentially fitted, as shown in Figure 11. Interestingly, the weight loss of CNR-DEDB showed a typical exponential process in the first 28 days, demonstrating that its long-term degradation could be controllable.

## 4. Conclusions

ENR was partially cyclically carbonated by the reaction of epoxy groups in the backbone of ENR with dioxide carbon under high pressure. The residual epoxy groups of the polymer were then clicked to the double terminal thiol groups of the novel cross-linking agent containing a boronic ester bond and a disulfide bond to introduce a self-healing function to the network. The physicochemical structure of cyclic carbonated natural rubber was characterized by means of FTIR, ^1^H NMR, and DSC. The formation of cyclic carbonate groups endowed the elastomer with biodegradability, which can be confirmed by the further biodegradation test. After the sample was immersed in PBS for 28 days, the biodegradation rate reached 22.37%. At the same time, the biodegradable rubber can be self-healed owing to the introduction of double dynamical covalent bonds. At a constant temperature of 60 °C, transesterification of boron ester bonds and disulfide exchange led to the movement of molecular chain segments, which enabled the cut samples to be re-crosslinked. The healing degree was as high as 82.87%.

## 5. Patents

Rentong Yu has the patent of “Preparation of biodegradable elastomers” issued by Hainan University; the patent number is CN113336930.

## Figures and Tables

**Table 1:** *T*_d-5%_ and *T*_d-max_ of ENR, CNR and CNR-DEDB.

Sample	*T*_d-5%_(°C)	*T*_d-max_(°C)
ENR	336.06	452.17
CNR	322.16	447.36
CNR-DEDB	363.96	464.32

## Data Availability

The data presented in this study are available on request from the corresponding author.
